
JOURNAL INFORMATION
==============================
NLM Title Abbreviation: Access Microbiol
Journal ID: Access Microbiol
NLM Journal ID: 101746927
Journal ID: acmi

Access Microbiology

EISSN: 2516-8290
Publisher: Microbiology Society

ARTICLE INFORMATION
==============================
PMCID: PMC7491926
PMID: 32974497
Article version: 1
Subjects: Oral

Abstracts from the International Meeting on Arboviruses and their Vectors 2019

Publication date: 2019
Electronic publication date: 2019 Dec 20
Volume: 1
Issue: 10
Electronic Location ID: 5
Copyright: © 2019 The Authors
Copyright year: 2019
License: This is an open-access article distributed under the terms of the Creative Commons Attribution License.
License URL: https://creativecommons.org/licenses/by/4.0/

==============================


Chikungunya virus recruits the ESCRT trafficking machinery at multiple steps during the intracellular life cycle

http://jmmcr.microbiologyresearch.org

Torii Shiho *
Orba Yasuko
Sasaki Michihito
Wada Yuji
Hobson-Peters Jody
Hall Roy
Sawa Hirofumi
Division of Molecular Pathobiology , Research Center for Zoonosis Control , Sapporo
Department of Pathology , National Institute of Infectious Diseases , Shinjuku
Australian Infectious Diseases Research Centre , School of Chemistry and Molecular Biosciences , The University of Queensland
Global Virus Network , Baltimore , USA
Global Institution for Collaborative Research and Education (GI-CoRE) , Sapporo , Japan
*Correspondence:Shiho TORII, torii@czc.hokudai.ac.jp

Novel flaviviruses of the ocean: Insights into the evolution and circulation of flaviviruses between marine invertebrate and vertebrate Hosts

http://jmmcr.microbiologyresearch.org

Parry Rhys *
Asgari Sassan
Australian Infectious Disease Research Centre , School of Biological Sciences , The University of Queensland
*Correspondence:Rhys Parry, r.parry@uq.edu.au

Modulation of arbovirus infection by mosquito saliva

http://jmmcr.microbiologyresearch.org

Lefteri Daniella A *
Pondeville Emilie
Bryden Steven R
Pingen Marieke
McKimmie Clive S
University of Leeds , Leeds , United Kingdom
University of Glasgow , Glasgow , United Kingdom
*Correspondence:Daniella Lefteri, bs11dl@leeds.ac.uk

Virus-host interactions for Bluetongue virus and characterization of a new NS3 function

http://jmmcr.microbiologyresearch.org

POURCELOT Marie *
Kundlacz Cindy
Caignard Grégory
Fablet Aurore
Moraes Rayane Amaral Da Silva
Léger Thibaut
Rijn Piet A. van
Zientara Stephan
Vitour Damien
ENVA , Maison-Alfort , France
INSERM , Lyon , France
INRA , Maison-Alfort , France
ANSES , Maison-Alfort , France
CNRS , PARIS CEDEX 13 , France
WBVR , Lelystad , Netherlands
*Correspondence:Marie POURCELOT, marie.pourcelot@vet-alfort.fr

Budding induces symmetry defects in flavivirus virions

http://jmmcr.microbiologyresearch.org

Kuhn Richard *
Therkelsen Matthew
Klose Thomas
Rossmann Michael
Purdue University , West Lafayette , USA
*Correspondence:Richard Kuhn, kuhnr@purdue.edu

Orthobunyavirus recruits host ESCRT machinery to Golgi compartments to achieve efficient viral particle production

http://jmmcr.microbiologyresearch.org

Barbosa Natalia *
Dias Marcos
Mendonca Leila
Schindler Michael
Arruda Eurico
Silva Luis da
University of Sao Paulo , Sao Paulo , Brazil
University of Cambridge , Cambridge , United Kingdom
University of Tuebingen , Tuebingen , Germany
*Correspondence:Natalia Barbosa, sbarbosanatalia@gmail.com

Developing a pseudotype model to test infectivity among different Zika virus mutants

http://jmmcr.microbiologyresearch.org

Jimenez Fernando Ruiz *
Olais Jose Humberto Perez
Ball Jonathan
University of Nottingham , Nottingham , United Kingdom
CINVESTAV , Mexico city , Mexico
*Correspondence: Fernando Ruiz Jimenez, mbxfr2@nottingham.ac.uk

Host genome depletion to determine the evolution, genetic diversity and transmission patterns of full genome sequences of African swine fever genotype IX from Uganda

http://jmmcr.microbiologyresearch.org

Filipe Ana Da Silva
Vattipally Sreenu B.
Mair Daniel
Ogweng Peter
Mayega Johnson
Muwanika Vincent
Palmarini Massimo
Biek Roman
Masembe Charles *
MRC-University of Glasgow Centre for Virus Research , Glasgow , United Kingdom
College of Natural Sciences , Makerere University , Kampala
Institute of Biodiversity , Animal Health and Comparative Medicine , University of Glasgow
*Correspondence: Charles Masembe, cmasembe@cns.mak.ac.ug

